# Gap Junctions Between Striatal D1 Neurons and Cholinergic Interneurons

**DOI:** 10.3389/fncel.2021.674399

**Published:** 2021-06-08

**Authors:** Yuqi Ren, Yang Liu, Minmin Luo

**Affiliations:** ^1^School of Life Sciences, Peking University, Beijing, China; ^2^Peking University-Tsinghua University-NIBS Joint Graduate Program, Beijing, China; ^3^National Institute of Biological Sciences, Beijing, China; ^4^School of Life Sciences, Tsinghua University, Beijing, China; ^5^Tsinghua-Peking Joint Center for Life Sciences, Tsinghua University, Beijing, China; ^6^Chinese Institute for Brain Research, Beijing, China; ^7^Tsinghua Institute of Multidisciplinary Biomedical Research, Beijing, China

**Keywords:** medium spiny neuron, tonically active neurons, electrical synapse, optogenetics, whole-cell patch, motor learning

## Abstract

The striatum participates in numerous important behaviors. Its principal projection neurons use GABA and peptides as neurotransmitters and interact extensively with interneurons, including cholinergic interneurons (ChIs) that are tonically active. Dissecting the interactions between projection neurons and ChIs is important for uncovering the role and mechanisms of the striatal microcircuits. Here, by combining several optogenetic tools with cell type-specific electrophysiological recordings, we uncovered direct electrical coupling between D1-type projection neurons and ChIs, in addition to the chemical transmission between these two major cell types. Optogenetic stimulation or inhibition led to bilateral current exchanges between D1 neurons and ChIs, which can be abolished by gap junction blockers. We further confirmed the presence of gap junctions through paired electrophysiological recordings and dye microinjections. Finally, we found that activating D1 neurons promotes basal activity of ChIs via gap junctions. Collectively, these results reveal the coexistence of the chemical synapse and gap junctions between D1 neurons and ChIs, which contributes to maintaining the tonically active firing patterns of ChIs.

## Introduction

As the main input nucleus of the basal ganglia, the striatum participates in motor control and goal-directed behavior (Abudukeyoumu et al., 2019). Dysfunctions of the basal ganglia are related to several neurological diseases, such as Parkinson’s disease and Huntington’s disease (Graybiel, 2000; Levine et al., 2011). Medium-sized spiny neurons (MSNs) comprise over 95% of the striatal cell population (Kreitzer, 2009). MSNs are typically classified into two subpopulations containing neurons expressing dopamine D1 receptor subtypes or dopamine D2 receptor subtypes, which project to the globus pallidus interna (GPi) and the substantia nigra pars reticularta (SNR) or the globus pallidus externa (GPe) (Gerfen, 1992; Lobo, 2009), respectively. Both types of projection neurons are GABAergic and inhibitory (Kreitzer, 2009). D1-MSNs also contain the peptide neurotransmitters dynorphin and substance P (SP, also known as tachykinin 1, Tac1), whereas D2-MSNs contain encephalin (Gerfen, 2000; Lobo et al., 2006). The remaining neurons consist of cholinergic interneurons (ChIs), and several different subtypes of GABAergic interneurons, such as NPY/SST/NOS interneurons, NPY-NGF (neurogliaform) interneurons, PV fast-spiking interneurons, TH interneurons, and CCK interneurons (Tepper et al., 2010; Ibanez-Sandoval et al., 2011; Assous et al., 2017; Munoz-Manchado et al., 2018; Tepper et al., 2018). ChIs and SST neurons have been reported to express neurokinin-1 receptors (NK1R) in the striatum (Tuluc et al., 2009; Wang and Angulo, 2011).

ChIs represent a small population of striatal neurons (1–2%) but exhibit broad arborizations in the striatum. ChIs exert a complex and powerful influence on the function of the striatum, such as co-releasing glutamate and acetylcholine to MSNs (Higley et al., 2011; Mamaligas and Ford, 2016), driving GABA release from dopaminergic terminals (Nelson et al., 2014), and triggering striatal DA release (Threlfell et al., 2012). ChIs are also referred to as tonically active neurons (TANs). They exhibit spontaneous tonic activity and respond to motivationally salient stimuli with a pause followed by a rebound increase, suggesting an important role of the basal tonic activation in their behavioral functions, including movement control and reward processing (Aosaki et al., 1995; Calabresi et al., 2000; Pisani et al., 2007; Goldberg and Reynolds, 2011; Cachope et al., 2012).

The exact microcircuitry between MSNs and ChIs remains unclear. MSNs might modulate the activity of ChIs through neurotransmitter release from chemical synapses. In addition to the chemical synaptic transmissions in the striatal microcircuits, electrical synapses (also known as gap junctions) are implicated in striatal neurons (Koós and Tepper, 1999; Venance et al., 2004; Cummings et al., 2008; English et al., 2011; Szydlowski et al., 2013). Gap junctions connect the cytosolic of two adjacent cells through a specialized channel constructed by two hemichannels (termed connexons). They allow the passage of small molecules up to 1,000 Da, which contributes to the generation of the synchronous activity (Kumar and Gilula, 1996; Sohl et al., 2005; Dere and Zlomuzica, 2012). It is unclear what kinds of cell types are electrically coupled in the striatum.

Here, we employed optogenetic tools to study the interaction between D1-MSNs and ChIs. In terms of optogenetic characters, channelrhodopsin-2 (ChR2) conducting cation-selective ion channel (Nagel et al., 2003; Boyden et al., 2005) and Lari with high-sequence homology to ArchT conducting proton (Han et al., 2011; Wu et al., 2019) were expressed in genetically identified D1-MSNs and ChIs. In addition to the chemical transmission, we found a slow current exchange between D1-MSNs and ChIs, which could be inhibited by gap junction blockers. In accordance with the optogenetic results, we also confirmed that D1-MSNs and ChIs are electrically coupled through paired electrophysiological recordings and dye microinjections. Furthermore, we found that activating D1-MSNs promotes the basal activity of ChIs, which can be decreased by bath application of gap junction blockers, indicating that D1-MSNs may contribute to the maintaining of the basal activity of ChIs through gap junctions.

## Materials and Methods

### Animals

All procedures were conducted with the approval of the Animal Care and Use Committee of the National Institute of Biological Sciences, Beijing, in accordance with governmental regulations of China. We used 6–12-week-old male or female mice. *ChAT-ChR2-EYFP* mice were gifts from G. Feng (MIT, Cambridge, MA, United States). *Tac1-IRES-Cre* (stock no: 021877), *Ai32* mice (stock no: 012569), and *SST-IRSE-Cre* mice (stock no: 013044) were obtained from Jackson Laboratory (Bar Harbor, ME, United States). *ChAT-Cre* mice were obtained from Mutant Mouse Research and Resource Center (Davis, CA, USA). Wildtype C57BL6/N mice were purchased from VitalRiver (Beijing, China).

### AAV Virus Preparation and Injections

AAV vectors carrying the fDIO-ChR2-EYFP, retro-Flp, DIO-mCherry, DIO-mGFP, and fDIO-Lari-mRbuy3 were packaged into serotype 2/9 vectors with titers ∼2 × 10^12^ particles/ml. AAV2-fDIO-ChR2-EYFP and AAV2-retro-Flp were purchased from Shanghai Taitool Bioscience (Shanghai, China). The pAAV-CAG-fDIO-Lari-mRbuy3 construct was a gift from Yulong Li’s lab. The pAAV-EF1a-DIO-hChR2 (H134R)-mCherry construct was a gift from Karl Deisseroth (Addgene plasmid #20297). We constructed these plasmids by replacing the coding region of ChR2-mCherry in the pAAV-EF1a-DIO-ChR2-mCherry plasmid with that of mGFP (Addgene plasmid #14757).

For AAV injections, adult mice were anesthetized with pentobarbital (i.p. 80 mg/kg) and then mounted to a stereotaxic apparatus. The craniotomy was conducted, and the recombinant AAV vectors were injected into the striatum (AP 0.74 mm; ML 2.0 mm; DV 2–4 mm) and the SNR (AP 34 mm; ML 1.3 mm; DV 4.4 mm).

### Brain Slice Preparation

We performed slice recordings as our laboratory previously described (Ren et al., 2011; Hu et al., 2012; Zhang et al., 2016). Briefly, adult mice were anesthetized with pentobarbital (i.p. 80 mg/kg) and then transcardially perfused with 5 ml ice-cold oxygenated perfusion solution. The perfusion solution contains reagents as follows (in millimolar): 225 sucrose, 119 NaCl, 2.5 KCl, 1 NaH_2_PO_4_, 4.9 MgCl_2_, 0.1 CaCl_2_, 26.2 NaHCO_3_, 1.25 glucose, 3 kynurenic acid, and 1 Na-ascorbate. Next, the mouse brains were dissected and transferred into ice-cold oxygenated slice solution. The slice solution contains reagents as follows (in millimolar): 110 choline chloride, 2.5 KCl, 0.5 CaCl_2_, 7 MgCl_2_, 1.3 NaH_2_PO_4_, 25 NaHCO_3_, 10 glucose, 1.3 Na-ascorbate, and 0.6 Na-pyruvate. The slice solution was adjusted to 305–315 Osm/kg using sucrose. Sections containing the striatum (200 μm) were cut with a vibratome (VT1200s, Leica Biosystems, Wetzlar, Germany) and then incubated in 33°C oxygenated artificial cerebrospinal fluid (ACSF) containing reagents as follows (in millimolar): 125 NaCl, 2.5 KCl, 2 CaCl_2_, 1.3 MgCl_2_, 1.3 NaH_2_PO_4_, 1.3 Na-ascorbate, 0.6 Na-pyruvate, 10 glucose, and 25 NaHCO_3_ (305–315 Osm/kg). All chemicals were purchased from Sigma-Aldrich (St. Louis, MO, United States).

### Patch Recording

The recording pipettes (3–4 MΩ) for whole-cell recordings and cell-attached recordings were pulled by P-1000 (Sutter Instrument, Novato, CA, United States). An internal solution filled in the recording pipettes contains reagents as follows (in millimolar): 130 K-gluconate, 10 HEPES, 0.6 EGTA, 5 KCl, 3 Na_2_ATP, 0.3 Na_3_GTP, 4 MgCl_2_, and 10 Na_2_-phosphocreatine (pH 7.2–7.4, 295–305 Osm/kg). Slice recordings were performed with MultiClamp700B (Molecular Devices, San Jose, CA, United States). The traces were low-pass filtered at 3 kHz and digitized at 10 kHz (Axon Digidata 1322A, Molecular Devices, San Jose, CA, United States). The electrophysiological data was analyzed with Clampfit 10.2 software (Molecular Devices, San Jose, CA, United States).

For optogenetic stimulation, an optical fiber (0.2 mm core diameter, NA = 0.22) linked to a 473 nm laser driver (MBL-III-473, Changchun New Industries Optoelectronics Technology Co., Changchun, China) or a 561 nm laser driver (MXL-W-561, Changchun New Industries Optoelectronics Technology Co., Changchun, China) was submerged in ACSF and placed ∼0.3 mm from the recording site. The light intensity reaching the brain tissue was 0.2–10 mW/mm^2^; 20 Hz–5 s (5 ms pulses) or 5 s continuous photostimulation was used in the whole procedure.

The drugs were applied through perfusion or local injection. Drugs used in the slice recordings were as follows: tetrodotoxin (TTX, 1 μM, Tocris Bioscience, Bristol, United Kingdom), a voltage-gated sodium channel blocker; picrotoxin (50 μM, Sigma-Aldrich, St. Louis, MO, United States), a blocker of GABA_A_ receptors; 6,7-dinitroquinoxaline-2,3-dione (DNQX, 10 μM, Sigma-Aldrich, St. Louis, MO, United States), an AMPA-type glutamate receptor blocker; 2-amino-5-phosphonovalerate (APV, 50 μM, Sigma-Aldrich, St. Louis, MO, United States), an NMDA-type glutamate receptor blocker; SR140333 (10 μM, Tocris Bioscience, Bristol, United Kingdom); FK888 hydrate (10 μM, Sigma-Aldrich, St. Louis, MO, United States) and L-703606 oxalate salt hydrate (100 nM, Sigma-Aldrich, St. Louis, MO, United States), NK1R blockers; carbenoxolone disodium (CBX, 200 μM, Tocris Bioscience, Bristol, United Kingdom) and quinine (200 μM, Sigma-Aldrich, St. Louis, MO, United States), gap junctions blockers; [Sar^9^, Met(O_2_)^11^]-substance P (1 μM, Tocris Bioscience, Bristol, United Kingdom), a selective NK1R agonist; and substance P (5 μM, Tocris Bioscience, Bristol, United Kingdom).

### Neurobiotin Microinjection

Neurobiotin (7.5 mg/ml; Vector Laboratories, Burlingame, CA, United States) was dissolved into the internal solution. We employed whole-cell recordings to the neuron with the pipette filled with Neurobiotin. After electrophysiological characterization, neurons were held for at least 30 min in current clamp and constantly injected with a depolarization current (500 ms, 500 pA, 1 Hz) to allow Neurobiotin filling. Subsequently, slices were fixed overnight in 4% paraformaldehyde at 4°C. Cy3-conjugated streptavidin (Jackson ImmunoResearch Laboratories. Inc., West Grove, PA, United States) was used to visualize the Neurobiotin signals.

### Dual-Patch Recordings

Dual whole-cell recordings were performed on pairs of a ChI neuron and a D1 neuron. The distance between two neurons never exceeded 50 μm. Current steps were applied to ChI (+800 pA and −800 pA, 1 s current steps) and then we recorded voltage responses in the D1 neuron. Voltage responses in the D1 neuron were analyzed with average traces from 10 sweeps.

### Immunohistochemistry

Adult mice were anesthetized with an overdose of pentobarbital and then transcardially perfused with 4% paraformaldehyde (PFA). Mouse brain was dissected and fixed in 4% PFA for 4 h. After cryoprotection in 30% sucrose, brain sections (35 μm) were cut on a cryostat microtome (Leica CM1950, Leica Biosystems, Wetzlar, Germany). After rinsing with PBS and 0.3% Triton-X in 0.1 M PBS (PBST), the brain sections were blocked with 2% (*w*/*v*) bovine serum (BSA) in PBST for 1 h. Then, the brain sections were incubated with primary antibodies at 4°C for 48 h and secondary antibodies at room temperature for 2 h. Images were collected using a Zeiss LSM510 Meta or Nikon A1 confocal microscope and analyzed using FIJI. The antibodies used were as follows: anti-choline acetyltransferase (1:200, goat, AB144P, MERCK, Kenilworth, NJ, United States), anti-NK1R (S8305, rabbit, 1:5,000, Sigma-Aldrich, St. Louis, MO, United States), goat anti-rabbit for NK1R (Cy3 and Alexa Fluor 488, Jackson ImmunoResearch Laboratories, Inc., West Grove, PA, United States), donkey anti-goat for choline acetyltransferase (Cy3 and Alexa Fluor 488, Jackson ImmunoResearch Laboratories, Inc., West Grove, PA, United States), and Cy3-streptavidin (1:500).

### Data Analysis

All results were expressed as mean ± SEM, and statistical significance was assessed with Student’s *t*-test. ^∗∗^*P* < 0.01; ^∗∗∗^*P* < 0.001; ^****^P < 0.0001; and n.s., not significant for all statistical analyses are presented in figures. The strength of the gap junction coupling was quantified by calculating the coupling coefficient (CC) and correlation coefficient. Coupling coefficient was the ratio between the voltage deflections in the post- and pre-electrical synapse, and 10 sweeps were averaged as follows: CC (%) = (Δ*V*_post__–_/ΔV_pre–_) × 100 (Hinckley and Ziskind-Conhaim, 2006). Correlation coefficient was calculated using MATLAB *corrcoef* function.

## Results

### Tetanic Photostimulation of Striatal D1 Neurons Induces a Typical Inward Current in ChIs

**FIGURE 1:**
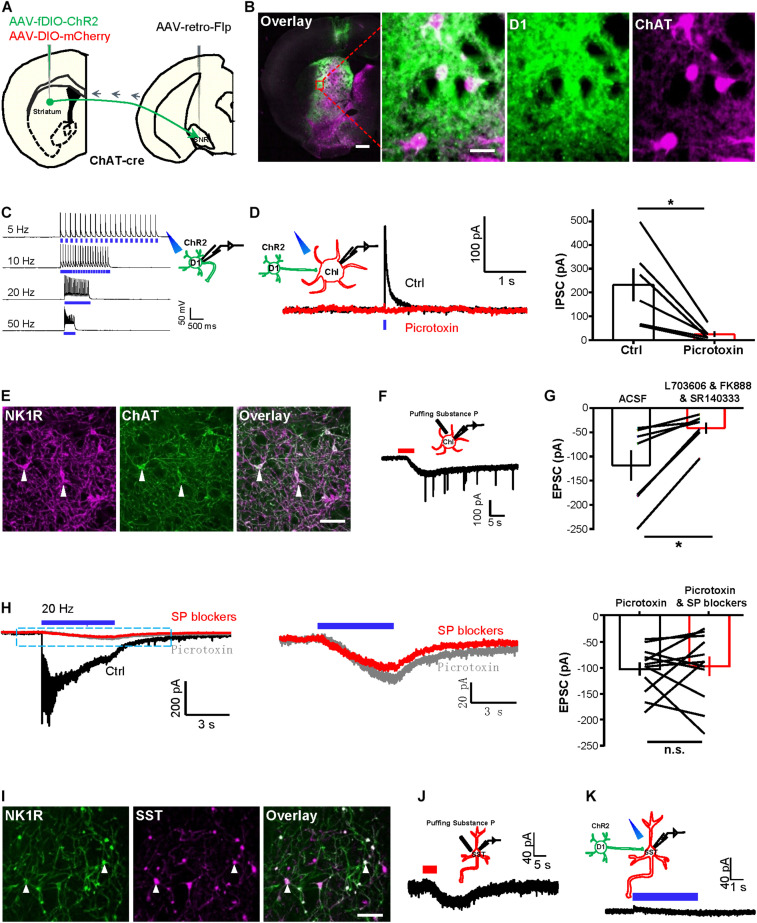
Tetanic photostimulation of D1 neurons induces an inward current in ChI that could not be inhibited by SP blockers. **(A)** Schematic representation showing the injection of AAV2/2-retro-hSyn-Flpo into the substantia nigra pars reticulata (SNR) and of AAV-fDIO-ChR2-EYFP and AAV-DIO-mCherry into the striatum of a *ChAT-Cre* mouse. **(B)** Images showing the expression of virus in the striatum (left panel), scale bar = 500 μm. The zoom-in view of the dashed rectangular area, showing the specific expression of ChR2 in D1 neurons and the specific expression of mCherry in ChIs (right panel). Green: D1 neurons with ChR2-EYFP; magenta: ChIs with mCherry. Scale bars = 50 μm. **(C)** A schematic diagram showing the method of whole-cell recording of the ChR2-expressing D1 neuron by 470 nm stimulation. Trains of brief laser pulse at 5, 10, 20, and 50 Hz (5 ms duration, 5 mW) produced precise firing of action potentials (*n* = 7 cells tested). **(D)** A schematic diagram and example traces (left) show brief optogenetic stimulation of ChR2-expressing D1 neurons produced a fast IPSC in ChI and the IPSC was inhibited by picrotoxin. The right panel shows group data. *p < 0.05 (paired *t*-test; *n* = 7 cells; ctrl: 231.6 ± 68.6 pA; mean ± SEM; picrotoxin: 25.2 ± 11.0 pA; recorded at −10 mV). **(E)** Images showing the specific expression of NK1R and ChAT in the striatum. White solid arrowheads indicated colocalized neurons. Scale bar = 100 μm. **(F)** A schematic diagram and example traces show that puffing 1 μM [Sar^9^, Met(O_2_)^11^]-substance P evoked inward currents from ChIs (*n* = 9 cells; peak amplitude: −75.1 ± 14.5 pA). **(G)** The group data show the effect of the SP blockers cocktail on the response of ChIs to the puffing of 5 μM substance P. *p < 0.05 (paired *t*-test; *n* = 7 cells; ctrl: −118.7 ± 31.5 pA; SP blockers: −41.4 ± 11.4 pA). **(H)** Example traces (left and middle) show the effects of picrotoxin and the SP blockers cocktail of L703606, FK888, and SR14033 on (left panel) the currents evoked by tetanic stimulation (20 Hz, 5 ms pulses, 5 s duration) of D1 neurons and recorded in a ChI. Summary data (right panel) show that the effects of SP blockers lack statistical significance. n.s., non-significant, *p* > 0.05 (paired *t*-test; *n* = 12 cells; ctrl: −102.5 ± 12.7 pA; SP blockers: −97.0 ± 18.6 pA at −65 mV). **(I)** Images show the expression of NK1R and SST in the striatum. White solid arrowheads indicated colocalized neurons. Scale bar = 200 μm. **(J)** A schematic diagram and example trace show the response of an SST neuron to the puffing of 1 μM [Sar^9^, Met(O_2_)^11^]-substance P (*n* = 5 cells; peak amplitude: −12.5 ± 3.2 pA). **(K)** A schematic diagram and example trace show the lack of response of an SST neuron in response to 5 s continuous optogenetic stimulation of D1 cells in TTX solution (*n* = 5 cells; peak amplitude: 4.8 ± 5.1 pA at −65 mV).

We used a dual-virus strategy to specifically label and manipulate striatal D1 neurons and ChIs (Gerfen, 1992; Guo et al., 2015; Li et al., 2018). Specifically, we injected retrograde transport-orientated adeno-associated virus (AAV) carrying recombinase flippase vector into the SNR and then injected AAV virus carrying flippase-dependent vector fDIO-ChR2-EYFP into the striatum to express ChR2 in D1 neurons projecting to the SNR (Figures 1A,B). As expected, we found that ChR2-EYFP-expressing D1 neurons projected to the SNR (Tervo et al., 2016). We also injected AAV-DIO-mCherry into the striatum of *ChAT-Cre* mice and thereby successfully labeled ChIs (Figures 1A,B). Whole-cell recordings in slice preparations revealed that trains of brief laser pulses at different frequencies induced precise firing of action potentials of D1 neurons (Figure 1C; *n* = 7 cells), confirming the validity of optogenetic stimulation.

We next examined the synaptic responses of ChIs to the photostimulation of ChR2-expressing D1 neurons. A brief light pulse induced fast inhibitory postsynaptic currents (IPSCs) in ChIs, and these were largely abolished upon the presence of the GABA_A_ blocker picrotoxin (Figure 1D). These findings collectively established that ChIs receive GABAergic synaptic transmission from D1 neurons.

Given that D1 neurons contain neuropeptide SP (Gerfen, 2000; Lobo et al., 2006), we asked whether activating D1 neural terminals induces the release of SP to striatal ChIs. NK1R (also known as TACR1 and the SP receptor) was colocalized with ChIs and SST neurons in the striatum (Tuluc et al., 2009; Wang and Angulo, 2011). Immunostaining against NK1R confirmed that ChIs expresses NK1R (Figure 1E). Whole-cell patch recordings showed that the slow currents evoked by puffing 1 μM [Sar^9^, Met(O_2_)^11^]-SP, a potent NK1R agonist (Blomeley and Bracci, 2008; Sosulina et al., 2015), exhibited a peak amplitude of −75.1 ± 14.5 pA (Figure 1F; mean ± SEM; *n* = 9 cells). Furthermore, the SP currents were inhibited (by 65%) upon perfusing a cocktail of SP blockers comprising SR140333 (Emonds-Alt et al., 1993; Huang et al., 2010), FK888 (Ferreira et al., 2005; Andrade et al., 2008), and L-703606 (Ikeda et al., 2003; Robinson et al., 2012; Figure 1G). These results thus demonstrate that the NK1Rs on ChIs are functional. Given that a brief pulse evoked fast IPSCs that were mediated through GABA_A_ receptors, we tested whether SP could be released by tetanic stimulation of D1 neurons. We found that a 20 Hz–5 s stimulation of D1 neurons induced slow inward currents on ChIs (Figure 1H, left panel). However, the application of SP blockers did not significantly reduce the inward currents (Figure 1H), and the remaining slow inward currents exhibited a mean amplitude of −97.0 ± 18.6 pA isolated with picrotoxin and SP blockers, suggesting that they were not secondary to the released SP. Similarly, we found that SST neurons express NK1R (Figure 1I) and were activated by puffing 1 μM [Sar^9^, Met(O_2_)^11^]-SP with a mean amplitude of −12.5 ± 3.2 pA (Figure 1J; *n* = 5 cells). However, tetanic stimulation (5 s continuous) of D1 neurons did not induce slow inward currents on SST neurons (Figure 1K). Together, these results revealed that tetanic photostimulation of striatal D1 neurons induces an inward current in ChI that could not be inhibited by SP blockers.

**FIGURE 2:**
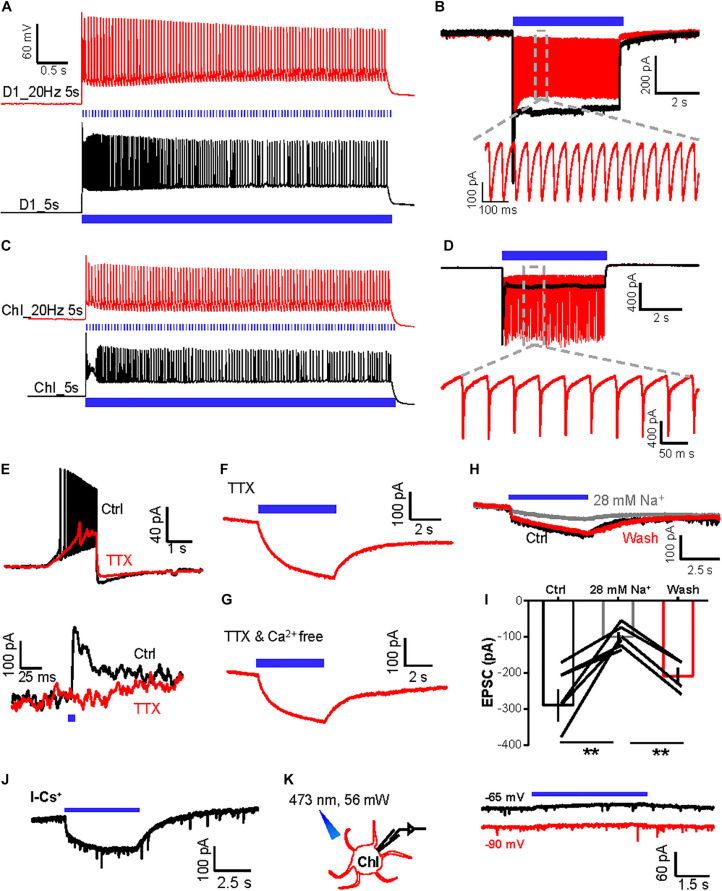
The slow inward currents induced by activation of D1 neurons are independent of action potential firing and extracellular Ca^2+^. **(A,B)** A schematic diagram showing the method of whole-cell recording of the ChR2-expressing D1 neuron by 470 nm stimulation in the current-clamp mode **(A)** and voltage-clamp mode **(B)**. Trains of tetanic stimulation laser pulse at 20 Hz–5 s (5 ms duration, 5 mW, red line) and 5 s continuous (black line) produced tetanic firing of action potentials **(A)** and inward currents **(B)** (*n* = 10 cells tested). **(C,D)** A schematic diagram showing the method of whole-cell recording of the ChR2-expressing ChI by 470 nm stimulation in the current-clamp recording mode **(C)** and voltage-clamp recording mode **(D)**. Trains of tetanic stimulation laser pulse at 20 Hz–5 s (5 ms duration, 5 mW, red line) and 5 s continuous (black line) produced tetanic firing of action potentials **(C)** and inward currents **(D)** (*n* = 6 cells tested). **(E)** Effect of TTX on the firing of action potentials following 100 pA inward current injection into a ChI (upper) and the fast IPSC evoked by a brief photostimulation of D1 neurons (*n* = 9 ChIs). **(F,G)** The slow inward current in a ChI evoked by 20 Hz–5 s stimulation of D1 neurons in the presence of TTX [**(F)**; *n* = 9 cells; peak amplitude: −170.2 ± 27.6 pA; at −90 mV] and TTX and Ca^2+^-free solution [**(G)**; *n* = 5 cells; peak amplitude: −219.0 ± 15.9 pA; at −90 mV]. **(H,I)** Example traces **(H)** and group data **(I)** show the effect of reducing extracellular Na^+^ concentration to 28 mM on the inward currents in the ChI in response to 5 s continuous optogenetic stimulation of the ChR2-expressing D1 neurons in the presence of TTX. **p < 0.01 (paired *t*-test; *n* = 7 cells; ctrl: −289.6 ± 43.4 pA; 28 mM Na^+^: −99.7 ± 12.6 pA; wash: −208.7 ± 22.9 pA). **(J)** In TTX solution, 5 s continuous optogenetic stimulation of D1 neurons continued to evoke a slow inward current in a ChI recorded with a Cs^+^-based internal solution (*n* = 6 cells; peak amplitude: −154.1 ± 66.4 pA). **(K)** 5 s continuous stimulation (470 nm, 56 mW power) produced a slow outward current in an mCherry-expressing ChI neuron held at −65 and −90 mV in the presence of TTX (*n* = 7 ChI cells; peak amplitude at −65 mV: 33.4 ± 9.5 pA; peak amplitude at −90 mV: 18.9 ± 9.5 pA).

We then studied the nature of the slow inward currents in ChIs in response to the tetanic stimulation of D1 neurons. We confirmed that 20 Hz–5 s stimulation of ChR2-expressing D1 neurons or ChIs generated precise firing of action potentials, and 5 s continuous stimulation of ChR2-expressing D1 neurons or ChIs generated action potential firings in the current-clamp recording mode and induced inward currents in the voltage-clamp recording mode (Figures 2A–D). TTX—a voltage-gated sodium channel blocker—significantly disrupted action potential firing of D1 neurons and inhibited the fast IPSCs on ChIs induced by a brief light pulse stimulation of D1 neurons (Figure 2E; *n* = 9 cells), indicating the requirement of action potentials to induce synaptic transmission. However, the slow inward currents of ChIs evoked by 20 Hz–5 s stimulation of D1 neurons were still detected when using the TTX solution or a TTX and nominally Ca^2+^-free extracellular solution, with peak amplitudes of −170.2 ± 27.6 pA (*n* = 9 cells) and −219.0 ± 15.9 pA (*n* = 5 cells), respectively (Figures 2F,G). These results suggest that the slow inward currents of ChIs are independent of action potential firing and of Ca^2+^ influx.

We tested whether changing the ion concentrations of ACSF affects the slow inward currents of ChIs. A low-concentration sodium solution (28 mM Na^+^) reversibly reduced the amplitudes of slow inward currents of ChIs evoked by 5 s continuous stimulation of ChR2-expressing D1 neurons (Figure 2H,I; *n* = 7 cells), demonstrating that sodium entry through ChR2 channels in D1 neurons is necessary for the generation of the slow inward currents of ChIs.

A recent study reported that light-driven temperature changes can affect striatal MSNs firing rates, specifically through inwardly rectifying potassium channels (Owen et al., 2019). We found that the slow inward currents were unaffected in ChIs recorded using a cesium-based internal solution, which excluded the role of potassium channels in the slow inward currents (Figure 2J; *n* = 6 cells; peak amplitude: −154.1 ± 66.4 pA) (Owen et al., 2019). Moreover, we labeled D1 neurons with GFP and then performed whole-cell recordings from ChIs with constant light delivery at 470 nm: light delivery induced a small outward current of ChIs rather than inward current (Figure 2K; *n* = 7 cells), excluding the role of light-driven temperature changes in our case. Collectively, these results reveal that tetanic stimulation of D1 neurons produces both fast GABAergic neurotransmission and a slow SP-independent current in ChIs. As the slow inward currents between D1 neurons and ChIs were independent of action potential firing and of Ca^2+^ influx and were not caused by light-driven temperature effect, we hypothesized that the slow currents of ChIs responding to light delivery could arise from ions influx of optogenetic channels through gap junction channels (Wang et al., 2014).

### Gap Junctions Connect D1 Neurons and ChIs

**FIGURE 3:**
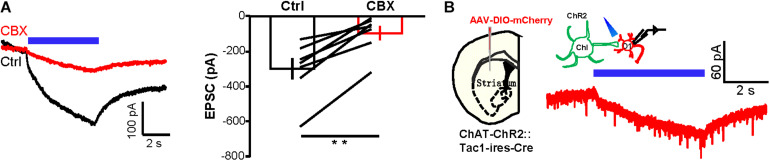
CBX substantially suppresses the current exchange between D1 neurons and ChIs. **(A)** Example traces and group data show the effect of CBX (200 μM) on the inward current of ChIs in response to 5 s continuous optogenetic stimulation of D1 neurons in the presence of TTX. **p < 0.01 (paired *t*-test; *n* = 7 cells; ctrl: −299.2 ± 61.5 pA; CBX: −95.7 ± 41.0 pA). **(B)** Schematics and example traces showing that 5 s continuous stimulation of ChIs evoked a slow inward current in a D1 neuron from a AAV-DIO-mCherry-injected *ChAT-ChR2-EYFP:Tac1-ires-Cre* mouse in the presence of TTX (*n* = 18 D1 cells; peak amplitude: −28.7 ± 3.3 pA).

We provided several additional lines of experimental evidence to demonstrate the existence of gap junctions between D1 neurons and ChIs. First, we tested the effect of the gap junction blocker CBX (Ross et al., 2000; Morita et al., 2007; Beck et al., 2008; Behrens et al., 2011; Benedikt et al., 2012; Manjarrez-Marmolejo and Franco-Pérez, 2016; Spray et al., 2019). CBX (200 μM) reduced the amplitudes of slow inward currents by 71.1% (Figure 3A; *n* = 8 cells). Second, we showed that the connections between D1 neurons and ChIs conduct bidirectional connection, a typical property of gap junction channels. We expressed ChR2 in ChIs and recorded from fluorescently labeled D1 neurons. Indeed, we found that there were small slow currents on D1 neurons during 5 s continuous activation of ChIs in TTX solution (Figure 3B; *n* = 18 cells; peak amplitude: −28.7 ± 3.3 pA).

**FIGURE 4:**
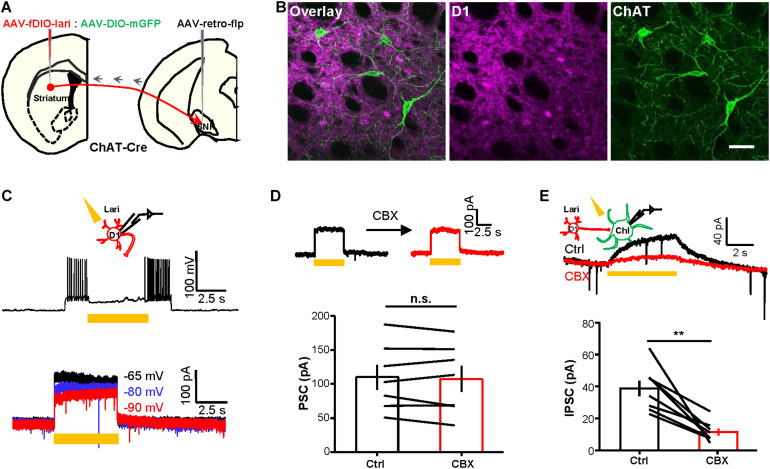
Optogenetically evoked outward currents in D1 neurons induce CBX-sensitive outward current in ChIs. **(A,B)** Schematics **(A)** and images **(B)** show the method and expression pattern of labeling D1 cells with Lari-mRuby3 and ChIs with mGFP. Scale bar = 50 μm. **(C)** 5 s continuous stimulation (561 nm, 10 mW) suppressed the action potential firing of a Lari-expressing D1 neuron and generated outward currents of various amplitudes at the holding potential of −65, −80, and −90 mV (*n* = 7 cells). **(D,E)** CBX (200 μM) did not affect the optogenetically evoked outward currents in Lari-expressing D1 neurons [**(D)**; n.s., non-significant; paired *t*-test; *n* = 7 cells; ctrl: 110.0 ± 18.4 pA; CBX: 107.5 ± 19.1 pA] but significantly suppressed the TTX-resistant outward currents recorded from nearby ChIs [**(E)**; **p < 0.01; paired *t*-test; *n* = 8 cells; ctrl: 38.7 ± 4.9 pA; CBX: 11.4 ± 2.2 pA].

Third, we used an optogenetic approach to demonstrate that inhibiting currents could also pass from D1 neurons to ChIs. We expressed Lari in D1 neurons and recorded from ChIs (Figures 4A,B). Lari is a light-driven outward proton pump and enables Lari-expressing neurons to be efficiently silenced in response to light stimulation (Wu et al., 2019). Neural action potentials of D1 neurons were effectively suppressed by 5 s continuous light (561 nm) under the current-clamp mode and generated outward currents in response to the different holding potentials in voltage-clamp recordings (Figure 4C; *n* = 7 cells), results collectively supporting that 561 nm laser stimulation reliably opens this proton pump. We then performed whole-cell recordings of ChIs during stimulation of Lari-expressing D1 neurons: whereas the outward currents in Lari-expressing D1 neurons were not obviously affected by CBX (Figure 4D; *n* = 7 cells), the outward currents of ChIs were inhibited by 67.7% in the presence of CBX (Figure 4E; *n* = 8 cells).

**FIGURE 5:**
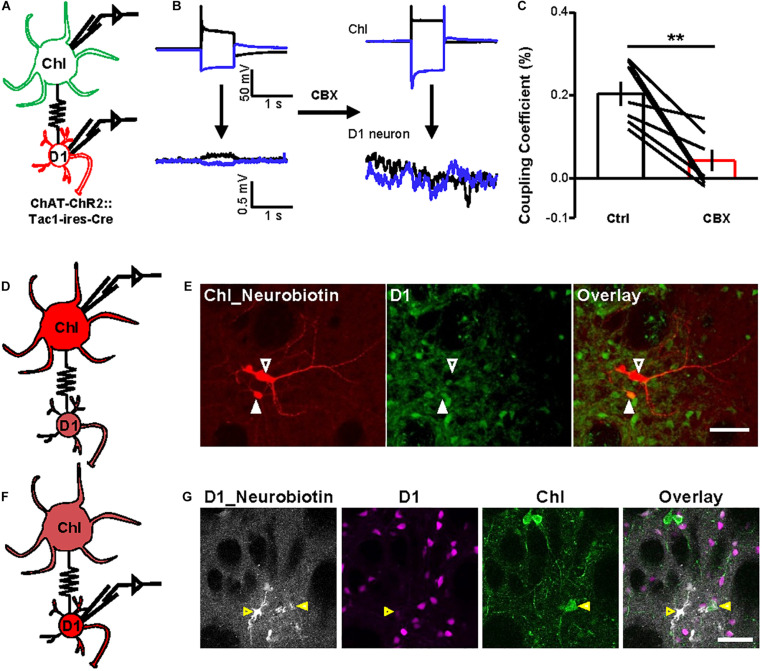
Dual-patch recording and dye microinjection reveal gap junctions between ChIs and D1 neurons. **(A)** A schematic diagram show the dual-patch recordings from a ChI–D1 neuron pair. **(B,C)** Current steps were applied to ChIs (−800 and + 800 pA, 1 s current steps) and then the voltage responses of D1 neuron were recorded (average traces from 10 sweeps). Example trace shows the blockade of electrical coupling by 200 μM CBX **(B)**. Summary data shows the coupling coefficient induced by depolarization of ChI was significantly reduced by 200 μM CBX **(C)**. **p < 0.01 (paired *t*-test; *n* = 7 coupled ChI–D1 pairs; ctrl: coupling coefficient by depolarization: 0.3 ± 0.03%; CBX: coupling coefficient by depolarization: 0.04 ± 0.02%). We also calculated the correlation coefficient of the coupled D1–ChIs pairs (ctrl: correlation coefficient by depolarization: 0.66 ± 0.04; CBX: correlation coefficient by depolarization: 0.24 ± 0.08; ctrl: correlation coefficient by hyperpolarization: 0.64 ± 0.03; CBX: correlation coefficient by hyperpolarization: 0.23 ± 0.09). **(D,E)** Schematics **(D)** and images **(E)** show that the Neurobiotin loaded into a ChI by whole-cell recording was detected in D1 neurons and other cell types. In **(E)**, red indicates Neurobiotin visualized with Cy3-streptavidin and green indicates mGFP in D1 neurons. Open arrowhead indicates a ChI filled with Neurobiotin and the solid arrowhead indicate Neurobiotin-labeled D1 cell (*n* = 8/10 ChIs; one ChI coupled with 2.5 ± 0.5 D1 neurons). Scale bar = 50 μm. **(F,G)** Schematics **(F)** and images **(G)** show that the Neurobiotin loaded into a D1 neuron by whole-cell recording was detected in ChI. In **(G)**, gray indicates Neurobiotin visualized with Alexa Fluor^®^ 647 streptavidin conjugates, magenta indicates mscarlet in D1 neurons, and green indicates EYFP in ChI. Open arrowhead indicates a D1 neuron filled with Neurobiotin and the solid arrowhead indicates Neurobiotin-labeled ChI (*n* = 2/20 D1 neurons). Scale bar = 50 μm.

Fourth, we conducted dual-patch recordings to confirm the presence of gap junctions between D1 neurons and ChIs. By labeling D1 neurons with DIO-mCherry and labeling ChIs with ChR2-EYFP in *ChAT-ChR2-EYFP:Tac1-ires-Cre* mice, we were able to record D1 neurons and ChI cell pairs simultaneously (Figure 5A). We applied a blocker cocktail comprising DNQX, APV, SR140333, and picrotoxin to isolate the gap junctions. Under current-clamp conditions, depolarization of the ChI by + 800 pA current injection induced a simultaneous depolarization of D1 neuron. About 44% ChI–D1 neuron pairs (*n* = 20/45 pairs) were found to be electrically coupled, and these coupled pairs displayed a mean coupling coefficient of 0.3% (Figure 5B). CBX significantly inhibited the coupling coefficient of the ChI–D1 cell pairs induced by depolarization of the ChI by 79.0% (Figures 5B,C; *n* = 7 pairs).

Finally, we injected small molecules to assess the occurrence of gap junctional communication between ChIs and D1 neurons. We loaded Neurobiotin into a ChI during whole-cell recordings and examined Neurobiotin diffusion into mGFP-expressing D1 neurons (Figure 5D): somata, dendrites, and axons of recorded ChIs were clearly labeled with Neurobiotin (Figure 5E; *n* = 8/10 ChIs). Each Neurobiotin-containing ChI showed tracer coupling to an average of 2.5 D1 neurons (Figure 5E). We also loaded Neurobiotin into a D1 neuron and observed Neurobiotin diffusion into ChIs (Figures 5F,G). These results thus collectively demonstrated that striatal D1 neurons and ChIs form gap junctions.

### Activating D1 Neurons Promotes Basal Activity of ChIs via Gap Junctions

**FIGURE 6:**
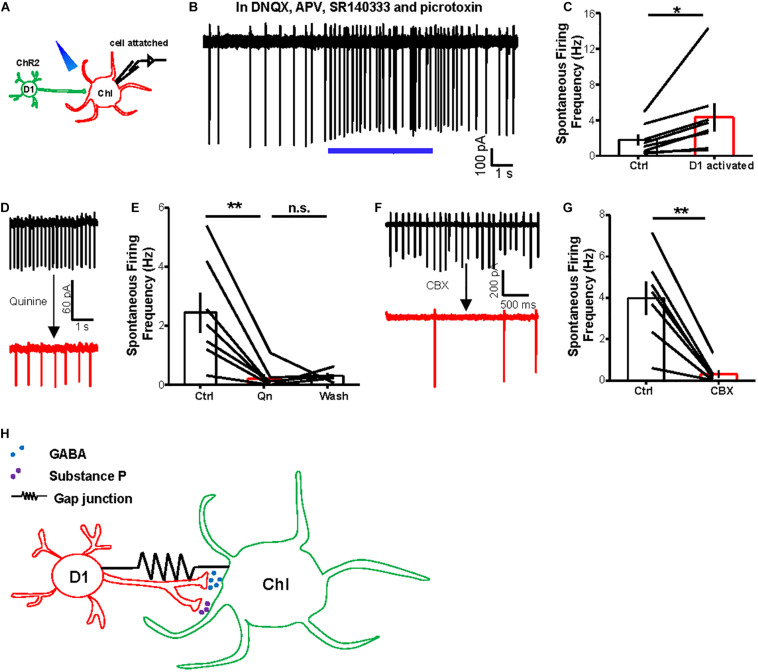
Activating D1 neurons promotes basal activity of ChIs via gap junctions. **(A)** Schematics show the method of cell-attached recording from a ChI and optogenetic stimulation of D1 cells (20 Hz–5 s light pulses). **(B,C)** An example trace **(B)** and summary data **(C)** show that optogenetic stimulation of D1 neurons enhanced the firing rates of ChIs in the presence of a cocktail solution comprising DNQX, APV, SR140333, and picrotoxin to block the release of major neurotransmitters in the striatum. In **(C)**, *p < 0.05 (paired *t*-test; *n* = 8; ctrl: 1.8 ± 0.6 Hz; activation: 4.3 ± 1.5 Hz). **(D,E)** Example traces **(D)** and the group data **(E)** show that quinine (200 μM) suppressed the basal firing rates of ChIs. **p < 0.01 (paired *t*-test; *n* = 7 cells; ctrl: 2.5 ± 0.7 Hz; quinine: 0.2 ± 0.2 Hz; wash: 0.3 ± 0.1 Hz). **(F,G)** The effect of CBX (200 μM) on the basal firing rates of a representative neuron **(F)** and the entire test group of ChIs **(G)**. **p < 0.01 (paired *t*-test; *n* = 7 cells; ctrl: 4.0 ± 0.8 Hz; CBX: 0.3 ± 0.2 Hz). **(H)** A diagram illustrates the interaction between D1 cells and ChIs via both chemical synapses and electrical synapses. D1 cells modulate the activity of ChIs through the release of GABA and substance P via chemical synapses. D1 cells and ChIs are also electrically coupled via gap junctions.

Previous studies have suggested that striatal ChIs exhibit spontaneous tonic activity (Aosaki et al., 1995; Goldberg and Reynolds, 2011). Given that ChIs receive inhibitory chemical synaptic inputs from D1 neurons, we hypothesized that the gap junctions between D1 neurons and ChIs may contribute to the basal activities of ChIs. Pursuing this, we performed cell-attached recordings from ChIs to assess whether D1–ChI neural gap junctions contribute to the basal activity of ChIs (Figure 6A). Stimulation of D1 neurons with 20 Hz–5 s trains increased ChI firing rates, from 1.8 to 4.3 Hz (ctrl: 1.8 ± 0.6 Hz; activation: 4.3 ± 1.5 Hz; *n* = 8 cells), upon the presence of a blocker cocktail solution comprising DNQX, APV, SR140333, and picrotoxin (Figures 6B,C), thus demonstrating that stimulating D1 neurons could increase the firing activity of ChIs through electrical coupling. In the slice preparation, ChIs exhibited tonic action potential firing, which is reminiscent of the tonic activation *in vivo* (Aosaki et al., 1995; Goldberg and Reynolds, 2011; Cachope et al., 2012). More importantly, the spontaneous firing rates of ChIs were inhibited by 90% following the bath application of gap junction blocker quinine (Figures 6D,E; *n* = 7 cells). Consistent with the effect of quinine, CBX also significantly reduced the basal activity of ChIs (Figures 6F,G; *n* = 7 cells). Collectively, these results suggest that the activity of D1 neurons drives enhanced basal activity of ChIs via gap junctions.

## Discussion

Combining optogenetics, paired electrophysiological recordings, and dye microinjections, here we report that striatal D1-MSNs interact with ChIs via both chemical synapses (through GABA release) and gap junctions (Figure 6H). Moreover, the gap junctions promote the basal activity of ChIs, the tonic activation pattern of which is considered important for ChI functions. Our results thus shed light on the mechanism of cholinergic functions in modulating striatal output signals.

We provide multiple lines of evidences to support the presence of gap junctions between D1-MSNs and ChIs. First, optogenetically induced inward currents or outward currents in D1 cells or ChIs bidirectionally pass between these two types of neurons. Second, this current exchange is suppressed by gap junction blockers but is resistant to various blockers of chemical neurotransmission. Third, dual whole-cell recordings of D1 cell and ChI pairs reveal that electrically evoked currents pass two individual cells and again this passage is blocked by gap junction blockers. Finally, dye microinjections in individual ChI spread to nearby cells, including D1 cells. These experimental results together provide compelling support to our conclusion that D1-MSNs and ChIs are electrically coupled, in addition to our data that confirm D1-MSNs release neurotransmitters onto ChIs. Interestingly, we do not observe such electrical coupling between D1 cells and somatostatin-expressing interneurons, which suggests cell type specificity for electrical coupling among striatal neurons.

It was previously held that gap junctions decrease markedly upon neural maturity (Pereda, 2014). However, in adult mammals, gap junctions still coexist with chemical synapses between neurons (Lefler et al., 2014; Lapato and Tiwari-Woodruff, 2018). This trend is especially evident in the striatum, where MSNs are reported to be both electrically and chemically coupled (Venance et al., 2004), although it had remained unknown whether projection neurons and interneurons are electrically coupled. Usually, electrical and dye coupling between neurons is often restricted to cells of the same class (Galarreta and Hestrin, 2001; Hormuzdi et al., 2004; Vandecasteele et al., 2007), but several examples of gap junctions between different types of neurons have been well documented. Previous studies suggest heterosynaptic electrotonic coupling between NGFs and THINs in the striatum (Assous et al., 2017; Tepper et al., 2018; Assous and Tepper, 2019). In the neocortex, there are electric synapses between SST interneurons and NPY-NGF interneurons, between SST interneurons and regular-spiking spiny stellate cells (putative excitatory neurons), and between fusiform interneurons and spiny stellate cells (Venance et al., 2000; Galarreta and Hestrin, 2001; Simon et al., 2005; Urban-Ciecko and Barth, 2016). Gap junctions between neurons and astrocytes have also been reported (Nedergaard, 1994; Alvarez-Maubecin et al., 2000). Taken together, although homologous electrical coupling is common, heterologous electrical coupling also exists, which may contribute to recruitment of neural network dependent on specific states and boost their efficacy in neural activity propagation.

Optogenetic manipulations enabled us to detect obvious electrical coupling between D1 cells and ChIs. Dual whole-cell recordings reveal that the coupling coefficients between D1-ChIs are small. There are two possibilities to explain why the coupling ratio of between D1 MSNs and ChIs is small. First, the coupling ratio will decrease following age increase (Meyer et al., 2002). For example, the coupling ratio in basket cell pairs detected in P14 is about 2.5%, while that detected in P42 brain slices is about 1.2% (Meyer et al., 2002). Most of previous studies (including Koós and Tepper, 1999) recorded from animals younger than 42 days old (Koós and Tepper, 1999; Meyer et al., 2002; Hormuzdi et al., 2004; Simon et al., 2005), while our study used adult mice. Second, coupling ratio of heterologous gap junctions is usually smaller than that of homologous gap junctions (Meyer et al., 2002; Simon et al., 2005). We speculate that multiple D1-MSNs may be connected to one ChI via gap junctions, a scenario that would enable amplification of ChI responses to electrical transmission during stimulation of optogenetic channel-expressing D1-MSNs. Moreover, comparing paired recording with current injection, our optogenetic stimulation protocol most likely activated a large number of D1 cells over a long period time. Therefore, ChIs may pool the effects of electrical transmission from multiple D1 neurons over a long period to exhibit a stronger excitatory response. Our use of multiple optogenetic tools allows us to observe clear electrical coupling between two neuron types, thus highlighting the advantage of cell type-specific optogenetics for studying electrical synapses.

Our observations provide a new mechanism to understand the tonically active firing patterns of ChIs. ChIs represent a key population of striatal interneurons and are involved in various aspects of signal processing in the striatum. Striatal ChIs *in vivo* show a spontaneous tonic firing activity and respond to the rewarding cues and associative motor learning-related stimuli with a transient pause followed by a rebound increase (Pisani et al., 2007). Striatal ChI firing drives spontaneous muscarinic activation in D1-MSNs (Mamaligas and Ford, 2016) and triggers GABA release from dopamine terminals to D1-MSNs (Nelson et al., 2014). Meanwhile, ChIs receive GABA transmission from D1-MSNs while responding to the dopamine inputs (Graybiel, 2000; Lahiri and Bevan, 2020). We find that in addition to the GABAergic, inhibitory transmission from D1-MSNs to ChIs, activating D1-MSNs promotes the basal activity of ChIs. Moreover, this outcome can be inhibited by gap junction blockers. Our results suggest that the gap junctions between D1-MSNs and ChIs may contribute to maintaining the tonically active firing patterns of ChIs, which have been implicated as key modulators of striatal microcircuits in the induction of synaptic plasticity, motor learning, and motor dysfunction (Abudukeyoumu et al., 2019).

This study also raises two questions that need to be further resolved. D1 MSNs have been reported to co-release GABA and SP onto ChIs (Bell et al., 1998; Govindaiah et al., 2010; Wang and Angulo, 2011; Francis et al., 2019). Consistently, we detected GABA release from D1-MSNs to ChIs via whole-cell recording. Francis et al. (2019) reported that high-frequency activation of D1-MSNs increased firing rates of ChIs and showed this can be inhibited by an SP blocker. In the present study, we applied whole-cell recordings to detect the currents induced by SP. Although SP blockers reduce slow inward currents in some ChIs induced by high-frequency activation of D1-MSNs, at the group level, they did not significantly suppress the inward currents, despite the presence of functional SP receptors on ChIs. SP release from D1 cells may also require certain modulation factors. For example, peptide neurokinin B release from habenula neurons is induced only after presynaptic excitation via GABA_B_ receptors (Zhang et al., 2016).

Moreover, the exact molecular identity of the gap junctions between D1 cells and ChIs remains to be dissected. In mammals, gap junction channels are encoded by a family of genes called “connexin (*Cx*),” which can be categorized as dependent on the molecular mass of the connexin protein. Different connexin subunits can selectively interact with each other to form homotypic and heterotypic channels (Mese et al., 2007). In adult rats, MSNs express Cx31.1, Cx32, Cx36, and Cx47 (Venance et al., 2004). Single-cell sequence data in the striatum show that D1-MSNs mainly express Cx33 and Cx45 and striatal interneurons express Cx29, Cx33, Cx36, and Cx45 (Gokce et al., 2016; Munoz-Manchado et al., 2018). Cx36 has been reported to localize to the striatal interneurons, and deletion of Cx36 reduces the spontaneous activities of MSNs (Cummings et al., 2008). These studies support the possibility of gap junctions between D1-ChIs. However, our preliminary results indicate that knocking down several individual connexin-encoding genes fails to abolish the electrical coupling between D1 cells and ChIs (data not shown), suggesting the gap junction channels between D1 neurons and ChIs comprise two or more connexin proteins (Koval et al., 2014). Such heterotypic composition has been demonstrated in the inferior olive and in the deep cerebellar nuclei (Dere and Zlomuzica, 2012).

In summary, this study provides the first demonstration of electrical coupling between D1-type projection neurons and ChIs. Moreover, such coupling contributes to the tonic activation pattern of cholinergic neurons. Our study suggests that a precise understanding of striatal microcircuits needs to integrate information both from chemical synapses and electrical synapses in a cell type-specific manner. Finally, our experimental approach of integrating optogenetics with pharmacological interventions and electrophysiology should facilitate the process of studying the presence of both types of synaptic connections in the nervous system.

## Data Availability Statement

The original contributions presented in the study are included in the article/supplementary material, further inquiries can be directed to the corresponding author/s.

## Ethics Statement

The animal study was reviewed and approved by the Animal Care and Use Committee of the National Institute of Biological Sciences, Beijing.

## Author Contributions

YR, YL, and ML designed the experiments. YR performed brain slice recordings, immunostaining, and surgeries. YL helped perform brain slice recordings. YR and ML analyzed the data and wrote the manuscript. All authors contributed to the article and approved the submitted version.

## Conflict of Interest

The authors declare that the research was conducted in the absence of any commercial or financial relationships that could be construed as a potential conflict of interest.

## References

[B1] Abudukeyoumu N. Hernandez-Flores T. Garcia-Munoz M. Arbuthnott G. W. (2019). Cholinergic modulation of striatal microcircuits. *Eur. J. Neurosci.* 49 604–622. 10.1111/ejn.13949 29797362 PMC6587740

[B2] Alvarez-Maubecin V. Fernando G. -H. Williams J. T. Bockstaele A. E. J. V. (2000). Functional coupling between neurons and glia. *J. Neurosci.* 20 4091–4098. 10.1523/jneurosci.20-11-04091.2000 10818144 PMC6772654

[B3] Andrade E. L. Luiz A. P. Ferreira J. Calixto J. B. (2008). Pronociceptive response elicited by TRPA1 receptor activation in mice. *Neuroscience* 152 511–520. 10.1016/j.neuroscience.2007.12.039 18272293

[B4] Aosaki T. Kimura M. Graybiel A. M. (1995). Temporal and spatial characteristics of tonically active neurons of the primate’s striatum. *J. Neurophysiol.* 73 1234–1252. 10.1152/jn.1995.73.3.1234 7608768

[B5] Assous M. Kaminer J. Shah F. Garg A. Koos T. Tepper J. M. (2017). Differential processing of thalamic information via distinct striatal interneuron circuits. *Nat. Commun.* 8:15860. 10.1038/ncomms15860 28604688 PMC5477498

[B6] Assous M. Tepper J. M. (2019). Excitatory extrinsic afferents to striatal interneurons and interactions with striatal microcircuitry. *Eur. J. Neurosci.* 49 593–603. 10.1111/ejn.13881 29480942 PMC6507406

[B7] Beck P. Odle A. Wallace-Huitt T. Skinner R. D. Garcia-Rill E. (2008). Modafinil increases arousal determined by P13 potential amplitude: an effect blocked by gap junction antagonists. *Sleep* 31 1647–1654. 10.1093/sleep/31.12.1647 19090320 PMC2603487

[B8] Behrens C. J. Ul Haq R. Liotta A. Anderson M. L. Heinemann U. (2011). Nonspecific effects of the gap junction blocker mefloquine on fast hippocampal network oscillations in the adult rat in vitro. *Neuroscience* 192 11–19. 10.1016/j.neuroscience.2011.07.015 21763755

[B9] Bell M. I. Richardson P. J. Lee K. (1998). Characterization of the mechanism of action of tachykinins in rat striatal cholinergic interneurons. *Neuroscience* 87 649–658. 10.1016/s0306-4522(98)00187-0 9758231

[B10] Benedikt J. Inyushin M. Kucheryavykh Y. V. Rivera Y. Kucheryavykh L. Y. Nichols C. G. (2012). Intracellular polyamines enhance astrocytic coupling. *Neuroreport* 23 1021–1025. 10.1097/WNR.0b013e32835aa04b 23076119 PMC3658138

[B11] Blomeley C. Bracci E. (2008). Substance P depolarizes striatal projection neurons and facilitates their glutamatergic inputs. *J. Physiol.* 586 2143–2155. 10.1113/jphysiol.2007.148965 18308827 PMC2465195

[B12] Boyden E. S. Zhang F. Bamberg E. Nagel G. Deisseroth K. (2005). Millisecond-timescale, genetically targeted optical control of neural activity. *Nat. Neurosci.* 8 1263–1268. 10.1038/nn1525 16116447

[B13] Cachope R. Mateo Y. Mathur B. N. Irving J. Wang H. L. Morales M. (2012). Selective activation of cholinergic interneurons enhances accumbal phasic dopamine release: setting the tone for reward processing. *Cell. Rep.* 2 33–41. 10.1016/j.celrep.2012.05.011 22840394 PMC3408582

[B14] Calabresi P. Centonze D. Gubellini P. Pisani A. Bernardi G. (2000). Acetylcholine-mediated modulation of striatal function. *Trends Neurosci.* 23 120–126. 10.1016/s0166-2236(99)01501-5 10675916

[B15] Cummings D. M. Yamazaki I. Cepeda C. Paul D. L. Levine M. S. (2008). Neuronal coupling via connexin36 contributes to spontaneous synaptic currents of striatal medium-sized spiny neurons. *J. Neurosci. Res.* 86 2147–2158. 10.1002/jnr.21674 18381762

[B16] Dere E. Zlomuzica A. (2012). The role of gap junctions in the brain in health and disease. *Neurosci. Biobehav. Rev.* 36 206–217. 10.1016/j.neubiorev.2011.05.015 21664373

[B17] Emonds-Alt X. Doutremepuich J.-D. Heaulme M. Neliat G. Santucci V. Steinberg R. (1993). In vitro and in vivo biological activities of SR140333, a novel potent non-peptide tachykinin NK1 receptor antagonist. *Eur. J. Pharmacol.* 250 403–413. 10.1016/0014-2999(93)90027-f 7509286

[B18] English D. F. Ibanez-Sandoval O. Stark E. Tecuapetla F. Buzsaki G. Deisseroth K. (2011). GABAergic circuits mediate the reinforcement-related signals of striatal cholinergic interneurons. *Nat. Neurosci.* 15 123–130. 10.1038/nn.2984 22158514 PMC3245803

[B19] Ferreira J. Triches K. M. Medeiros R. Calixto J. B. (2005). Mechanisms involved in the nociception produced by peripheral protein kinase c activation in mice. *Pain* 117 171–181. 10.1016/j.pain.2005.06.001 16099101

[B20] Francis T. C. Yano H. Demarest T. G. Shen H. Bonci A. (2019). High-Frequency Activation of Nucleus Accumbens D1-MSNs Drives Excitatory Potentiation on D2-MSNs. *Neuron* 103:432–444.e3. 10.1016/j.neuron.2019.05.031 31221559 PMC6712577

[B21] Galarreta M. Hestrin S. (2001). Electrical synapses between GABA-releasing interneurons. *Nat. Rev. Neurosci.* 2 425–433. 10.1038/35077566 11389476

[B22] Gerfen C. R. (1992). The neostriatal mosaic: multiple levels of compartmental organization in the basal ganglia. *Ann. Rev. Neurosci.* 15 285–320. 10.1146/annurev.ne.15.030192.001441 1575444

[B23] Gerfen C. R. (2000). Molecular effects of dopamine on striatal-projection pathways. *Trends Neurosci.* 23 S64–70. 11052222 10.1016/s1471-1931(00)00019-7

[B24] Gokce O. Stanley G. M. Treutlein B. Neff N. F. Camp J. G. Malenka R. C. (2016). Cellular Taxonomy of the Mouse Striatum as Revealed by Single-Cell RNA-Seq. *Cell. Rep.* 16 1126–1137. 10.1016/j.celrep.2016.06.059 27425622 PMC5004635

[B25] Goldberg J. A. Reynolds J. N. J. (2011). Spontaneous firing and evoked pauses in the tonically active cholinergic interneurons of the striatum. *Neuroscience* 198 27–43. 10.1016/j.neuroscience.2011.08.067 21925242

[B26] Govindaiah G. Wang Y. Cox C. L. (2010). Substance P selectively modulates GABA(A) receptor-mediated synaptic transmission in striatal cholinergic interneurons. *Neuropharmacology* 58 413–422. 10.1016/j.neuropharm.2009.09.011 19786036

[B27] Graybiel A. M. (2000). The basal ganglia. *Curr. Biol.* 10 R509–11. 10899013 10.1016/s0960-9822(00)00593-5

[B28] Guo Q. Wang D. He X. Feng Q. Lin R. Xu F. (2015). Whole-brain mapping of inputs to projection neurons and cholinergic interneurons in the dorsal striatum. *PLoS One* 10:e0123381. 10.1371/journal.pone.0123381 25830919 PMC4382118

[B29] Han X. Chow B. Y. Zhou H. Klapoetke N. C. Chuong A. Rajimehr R. (2011). A high-light sensitivity optical neural silencer: development and application to optogenetic control of non-human primate cortex. *Front. Syst. Neurosci.* 5:18. 10.3389/fnsys.2011.00018 21811444 PMC3082132

[B30] Higley M. J. Gittis A. H. Oldenburg I. A. Balthasar N. Seal R. P. Edwards R. H. (2011). Cholinergic Interneurons Mediate Fast VGluT3-Dependent Glutamatergic Transmission in the Striatum. *PLoS One* 6:e19155. 10.1371/journal.pone.0019155 21544206 PMC3081336

[B31] Hinckley C. A. Ziskind-Conhaim L. (2006). Electrical Coupling between Locomotor-Related Excitatory Interneurons in the Mammalian Spinal Cord. *J. Neurosci.* 26 8477–8483. 10.1523/jneurosci.0395-06.2006 16914672 PMC6674344

[B32] Hormuzdi S. G. Filippov M. A. Mitropoulou G. Monyer H. Bruzzone R. (2004). Electrical synapses: a dynamic signaling system that shapes the activity of neuronal networks. *Biochim. Biophys. Acta* 1662 113–137. 10.1016/j.bbamem.2003.10.023 15033583

[B33] Hu F. Ren J. Zhang J. E. Zhong W. Luo M. (2012). Natriuretic peptides block synaptic transmission by activating phosphodiesterase 2A and reducing presynaptic PKA activity. *Proc. Natl. Acad. Sci. U. S. A.* 109 17681–17686. 10.1073/pnas.1209185109 23045693 PMC3491473

[B34] Huang W. Q. Wang J. G. Chen L. Wei H. J. Chen H. (2010). SR140333 counteracts NK-1 mediated cell proliferation in human breast cancer cell line T47D. *J. Exp. Clin. Cancer Res.* 29:55. 10.1186/1756-9966-29-55 20497542 PMC2890547

[B35] Ibanez-Sandoval O. Tecuapetla F. Unal B. Shah F. Koos T. Tepper J. M. (2011). A novel functionally distinct subtype of striatal neuropeptide Y interneuron. *J. Neurosci.* 31 16757–16769. 10.1523/JNEUROSCI.2628-11.2011 22090502 PMC3236391

[B36] Ikeda H. Heinke B. Ruscheweyh R. Sandkühler J. (2003). Synaptic plasticity in spinal lamina I projection neurons that mediate hyperalgesia. *Science* 299 1237–1240. 10.1126/science.1080659 12595694

[B37] Koós T. Tepper A. J. M. (1999). Inhibitory control of neostriatal projection neurons by GABAergic interneurons. *Nat. Neurosci.* 2 467–472. 10.1038/8138 10321252

[B38] Koval M. Molina S. A. Burt J. M. (2014). Mix and match: investigating heteromeric and heterotypic gap junction channels in model systems and native tissues. *FEBS Lett.* 588 1193–1204. 10.1016/j.febslet.2014.02.025 24561196 PMC3992227

[B39] Kreitzer A. C. (2009). Physiology and pharmacology of striatal neurons. *Ann. Rev. Neurosci.* 32 127–147. 10.1146/annurev.neuro.051508.135422 19400717

[B40] Kumar N. M. Gilula N. B. (1996). The gap junction communication channel. *Cell* 84 381–388. 10.1016/s0092-8674(00)81282-9 8608591

[B41] Lahiri A. K. Bevan M. D. (2020). Dopaminergic Transmission Rapidly and Persistently Enhances Excitability of D1 Receptor-Expressing Striatal Projection Neurons. *Neuron* 106:277–290.e6. 10.1016/j.neuron.2020.01.028 32075716 PMC7182485

[B42] Lapato A. S. Tiwari-Woodruff S. K. (2018). Connexins and pannexins: at the junction of neuro-glial homeostasis & disease. *J. Neurosci. Res.* 96 31–44. 10.1002/jnr.24088 28580666 PMC5749981

[B43] Lefler Y. Yarom Y. Uusisaari M. Y. (2014). Cerebellar inhibitory input to the inferior olive decreases electrical coupling and blocks subthreshold oscillations. *Neuron* 81 1389–1400. 10.1016/j.neuron.2014.02.032 24656256

[B44] Levine M. S. Fisher Y. E. André V. M. (2011). Altered Balance of Activity in the Striatal Direct and Indirect Pathways in Mouse Models of Huntington’s Disease. *Front. Syst. Neurosci.* 5:46. 10.3389/fnsys.2011.00046 21720523 PMC3118454

[B45] Li Y. Zeng J. Zhang J. Yue C. Zhong W. Liu Z. (2018). Hypothalamic Circuits for Predation and Evasion. *Neuron* 97 911–924.e5. 10.1016/j.neuron.2018.01.005 29398361

[B46] Lobo M. K. (2009). Molecular Profiling of Striatonigral and Striatopallidal Medium Spiny Neurons past, present, and future. *Int. Rev. Neurobiol.* 89 1–35. 10.1016/s0074-7742(09)89001-6 19900613

[B47] Lobo M. K. Karsten S. L. Gray M. Geschwind D. H. Yang X. W. (2006). FACS-array profiling of striatal projection neuron subtypes in juvenile and adult mouse brains. *Nat. Neurosci.* 9 443–452. 10.1038/nn1654 16491081

[B48] Mamaligas A. A. Ford C. P. (2016). Spontaneous Synaptic Activation of Muscarinic Receptors by Striatal Cholinergic Neuron Firing. *Neuron* 91 574–586. 10.1016/j.neuron.2016.06.021 27373830 PMC5234077

[B49] Manjarrez-Marmolejo J. Franco-Pérez J. (2016). Gap junction blockers: an overview of their effects on induced seizures in animal models. *Curr. Neuropharmacol.* 14 759–771. 10.2174/1570159x14666160603115942 27262601 PMC5050393

[B50] Mese G. Richard G. White T. W. (2007). Gap junctions: basic structure and function. *J. Invest. Dermatol.* 127 2516–2524. 10.1038/sj.jid.5700770 17934503

[B51] Meyer A. H. Katona I. Blatow M. Rozov A. Monyer H. (2002). In vivo labeling of parvalbumin-positive interneurons and analysis of electrical coupling in identified neurons. *J. Neurosci.* 22 7055–7064. 10.1523/jneurosci.22-16-07055.2002 12177202 PMC6757887

[B52] Morita M. Saruta C. Kozuka N. Okubo Y. Itakura M. Takahashi M. (2007). Dual regulation of astrocyte gap junction hemichannels by growth factors and a pro-inflammatory cytokine via the mitogen-activated protein kinase cascade. *Glia* 55 508–515. 10.1002/glia.20471 17211868

[B53] Munoz-Manchado A. B. Bengtsson Gonzales C. Zeisel A. Munguba H. Bekkouche B. Skene N. G. (2018). Diversity of Interneurons in the Dorsal Striatum Revealed by Single-Cell RNA Sequencing and PatchSeq. *Cell. Rep.* 24 2179–2190.e7. 10.1016/j.celrep.2018.07.053 30134177 PMC6117871

[B54] Nagel G. Szellas T. Huhn W. Kateriya S. Adeishvili N. Berthold P. (2003). Channelrhodopsin-2, a directly light-gated cation-selective membrane channel. *Proc. Natl. Acad. Sci. U. S. A.* 100 13940–13945. 10.1073/pnas.1936192100 14615590 PMC283525

[B55] Nedergaard M. (1994). Direct signaling from astrocytes to neurons in cultures of mammalian brain cells. *Science* 263 1768–1771. 10.1126/science.8134839 8134839

[B56] Nelson A. B. Hammack N. Yang C. F. Shah N. M. Seal R. P. Kreitzer A. C. (2014). Striatal cholinergic interneurons Drive GABA release from dopamine terminals. *Neuron* 82 63–70. 10.1016/j.neuron.2014.01.023 24613418 PMC3976769

[B57] Owen S. F. Liu M. H. Kreitzer A. C. (2019). Thermal constraints on in vivo optogenetic manipulations. *Nat. Neurosci.* 22 1061–1065. 10.1038/s41593-019-0422-3 31209378 PMC6592769

[B58] Pereda A. E. (2014). Electrical synapses and their functional interactions with chemical synapses. *Nat. Rev. Neurosci.* 15 250–263. 10.1038/nrn3708 24619342 PMC4091911

[B59] Pisani A. Bernardi G. Ding J. Surmeier D. J. (2007). Re-emergence of striatal cholinergic interneurons in movement disorders. *Trends Neurosci.* 30 545–553. 10.1016/j.tins.2007.07.008 17904652

[B60] Ren J. Qin C. Hu F. Tan J. Qiu L. Zhao S. (2011). Habenula “cholinergic” neurons co-release glutamate and acetylcholine and activate postsynaptic neurons via distinct transmission modes. *Neuron* 69 445–452. 10.1016/j.neuron.2010.12.038 21315256

[B61] Robinson J. E. Fish E. W. Krouse M. C. Thorsell A. Heilig M. Malanga C. J. (2012). Potentiation of brain stimulation reward by morphine: effects of neurokinin-1 receptor antagonism. *Psychopharmacology* 220 215–224. 10.1007/s00213-011-2469-z 21909635 PMC3484369

[B62] Ross F. Gwyn P. Spanswick D. Davies S. J. N. (2000). Carbenoxolone depresses spontaneous epileptiform activity in the CA1 region of rat hippocampal slices. *Neuroscience* 100 789–796. 10.1016/s0306-4522(00)00346-8 11036212

[B63] Simon A. Olah S. Molnar G. Szabadics J. Tamas G. (2005). Gap-junctional coupling between neurogliaform cells and various interneuron types in the neocortex. *J. Neurosci.* 25 6278–6285. 10.1523/JNEUROSCI.1431-05.2005 16000617 PMC6725286

[B64] Sohl G. Maxeiner S. Willecke K. (2005). Expression and functions of neuronal gap junctions. *Nat. Rev. Neurosci.* 6 191–200. 10.1038/nrn1627 15738956

[B65] Sosulina L. Strippel C. Romo-Parra H. Walter A. L. Kanyshkova T. Sartori S. B. (2015). Substance P excites GABAergic neurons in the mouse central amygdala through neurokinin 1 receptor activation. *J. Neurophysiol.* 114 2500–2508. 10.1152/jn.00883.2014 26334021 PMC4620133

[B66] Spray D. C. Iglesias R. Shraer N. Suadicani S. O. Belzer V. Hanstein R. (2019). Gap junction mediated signaling between satellite glia and neurons in trigeminal ganglia. *Glia* 67 791–801. 10.1002/glia.23554 30715764 PMC6506223

[B67] Szydlowski S. N. Pollak Dorocic I. Planert H. Carlen M. Meletis K. Silberberg G. (2013). Target selectivity of feedforward inhibition by striatal fast-spiking interneurons. *J. Neurosci.* 33 1678–1683. 10.1523/JNEUROSCI.3572-12.2013 23345240 PMC6618742

[B68] Tepper J. M. Koos T. Ibanez-Sandoval O. Tecuapetla F. Faust T. W. Assous M. (2018). Heterogeneity and Diversity of Striatal GABAergic Interneurons: update 2018. *Front. Neuroanat.* 12:91. 10.3389/fnana.2018.00091 30467465 PMC6235948

[B69] Tepper J. M. Tecuapetla F. Koos T. Ibanez-Sandoval O. (2010). Heterogeneity and diversity of striatal GABAergic interneurons. *Front. Neuroanat.* 4:150. 10.3389/fnana.2010.00150 21228905 PMC3016690

[B70] Tervo D. G. Hwang B. Y. Viswanathan S. Gaj T. Lavzin M. Ritola K. D. (2016). A Designer AAV Variant Permits Efficient Retrograde Access to Projection Neurons. *Neuron* 92 372–382. 10.1016/j.neuron.2016.09.021 27720486 PMC5872824

[B71] Threlfell S. Lalic T. Platt N. J. Jennings K. A. Deisseroth K. Cragg S. J. (2012). Striatal dopamine release is triggered by synchronized activity in cholinergic interneurons. *Neuron* 75 58–64. 10.1016/j.neuron.2012.04.038 22794260

[B72] Tuluc F. Lai J. P. Kilpatrick L. E. Evans D. L. Douglas S. D. (2009). Neurokinin 1 receptor isoforms and the control of innate immunity. *Trends Immunol.* 30 271–276. 10.1016/j.it.2009.03.006 19427266

[B73] Urban-Ciecko J. Barth A. L. (2016). Somatostatin-expressing neurons in cortical networks. *Nat. Rev. Neurosci.* 17 401–409. 10.1038/nrn.2016.53 27225074 PMC5635659

[B74] Vandecasteele M. Deniau J. M. Glowinski J. Venance L. (2007). Electrical synapses in basal ganglia. *Rev. Neurosci.* 18 15–35. 10.1515/revneuro.2007.18.1.15 17405449

[B75] Venance L. Glowinski J. Giaume C. (2004). Electrical and chemical transmission between striatal GABAergic output neurones in rat brain slices. *J. Physiol.* 559 215–230. 10.1113/jphysiol.2004.065672 15235091 PMC1665072

[B76] Venance L. Rozov A. Blatow M. Burnashev N. Feldmeyer D. Monyer H. (2000). Connexin expression in electrically coupled postnatal rat brain neurons. *Proc. Natl. Acad. Sci. U. S. A.* 97 10260–10265. 10.1073/pnas.160037097 10944183 PMC27858

[B77] Wang J. Angulo J. A. (2011). Methamphetamine induces striatal neurokinin-1 receptor endocytosis primarily in somatostatin/NPY/NOS interneurons and the role of dopamine receptors in mice. *Synapse* 65 300–308. 10.1002/syn.20848 20730802 PMC2998568

[B78] Wang K. Gong J. Wang Q. Li H. Cheng Q. Liu Y. (2014). Parallel pathways convey olfactory information with opposite polarities in Drosophila. *Proc. Natl. Acad. Sci. U. S. A.* 111 3164–3169. 10.1073/pnas.1317911111 24516124 PMC3939862

[B79] Wu L. Dong A. Dong L. Wang S. Q. Li Y. (2019). PARIS, an optogenetic method for functionally mapping gap junctions. *ELife* 8:e43366. 10.7554/eLife.43366 30638447 PMC6396999

[B80] Zhang J. Tan L. Ren Y. Liang J. Lin R. Feng Q. (2016). Presynaptic Excitation via GABAB Receptors in Habenula Cholinergic Neurons Regulates Fear Memory Expression. *Cell* 166 716–728. 10.1016/j.cell.2016.06.026 27426949

